# Primary stability of the Activ L® intervertebral disc prosthesis in cadaver bone and comparison of the keel and spike anchoring concept

**DOI:** 10.1186/s12891-021-04544-7

**Published:** 2021-11-08

**Authors:** Christoph von Schulze Pellengahr, Wolfram Teske, Saurabh Kapoor, Alexander Klein, Bernd Wegener, Andreas Büttner, Matthias Lahner

**Affiliations:** 1 Department of Orthopaedic Surgery, Agaplesion Ev. Bathildis Hospital, Agaplesion Ev. Bathildis Krankenhaus, Maulbeerallee 4, 31812 Bad Pyrmont, Germany; 2 Clinic of Orthopaedic Surgery, Ruhr University Bochum, Bad Oeynhausen, Germany; 3 Center of Orthopaedic and Trauma Surgery, St.-Josef-Hospital Hagen, Hagen, Germany; 4 The Centre for Spinal Studies and Surgery, Queens Medical Centre, Nottingham, UK; 5 Clinic of Orthopaedic Surgery, Klinikum Großhadern, Ludwig-Maximilians University Munich, Munich, Germany; 6 Institute of Forensic Medicine, University of Rostock, Rostock, Germany; 7 Joint Center Hilden, Hilden, Germany

**Keywords:** Primary stability of intervertebral disc prosthesis, Micromotions, Aesculap ActivL, Anchoring concept, Keel, Spikes

## Abstract

**Background:**

High primary stability is the key prerequisite for safe osseointegration of cementless intervertebral disc prostheses. The aim of our study was to determine the primary stability of intervertebral disc prostheses with two different anchoring concepts – keel and spike anchoring.

**Methods:**

Ten ActivL intervertebral disc prostheses (5 x keel anchoring, 5 x spike anchoring) implanted in human cadaver lumbar spine specimens were tested in a spine movement simulator. Axial load flexion, extension, left and right bending and axial rotation motions were applied on the lumbar spine specimens through a defined three-dimensional movement program following ISO 2631 and ISO/CD 18192-1.3 standards. Tri-dimensional micromotions of the implants were measured for both anchor types and compared using Student’s T-test for significance after calculating 95 % confidence intervals.

**Results:**

In the transverse axis, the keel anchoring concept showed statistically significant (*p* < 0.05) lower mean values of micromotions compared to the spike anchoring concept. The highest micromotion values for both types were observed in the longitudinal axis. In no case the threshold of 200 micrometers was exceeded.

**Conclusions:**

Both fixation systems fulfill the required criteria of primary stability. Independent of the selected anchorage type an immediate postoperative active mobilization doesn’t compromise the stability of the prostheses.

## Background

In recent decades, several surgical methods (e.g. dorsolateral spinal fusion, dorsoventral spinal fusion, ventral spinal fusion, disc arthroplasties) were developed to treat degenerative pathologies of intervertebral disc and the bony vertebra. In contrast to spinal fusion techniques, the implantation of an artificial disc is intended to maintain the segmental mobility and to avoid an adjacent level disease.

Low back pain is very often related to intervertebral disc degeneration. As a consequence, there is increased medical interest for a successful treatment of disc diseases such as the use of artificial discs. This surgical treatment aims at restoring height of the segment and improving its biomechanical function.

The development of cementless implants in the last years has led to different anchoring concepts. The micromotion at the implant–bone interface has a significant effect on the primary stability of the prosthesis and can be determinant for obtaining long-term stability through adequate osseointegration.

No consensus yet exists regarding how much micromotion is required to achieve osteointegration. To date, no specific criteria for adequate micromotions have been reported for the primary stability of artificial discs in human. In vivo dog experiments indicated that increased micromotions can adversely affect osseointegration and suggested a threshold of 150 μm based on observations of connective tissue formation for micromotions above that limit [1]. This is slightly lower than limit of 200 μm for the osseointegration of cementless implants given by Pitto [2].

In this study, primary stability was tested experimentally for two different anchoring concepts (available are keel or spikes). Human lumbar spine specimens were subjected to simulated physiological cyclic motion in three planes under axial loading with the disc prothesis in place. Our experimental model with fresh frozen human specimens for biomechanical testing of the spine has long been established and described repeatedly in the literature [3–7].

Our hypotheses were two-fold:

• Both artificial discs tested in physiological motion patterns stay anchored to bone within the limit of 200 μm micromotion;
• Differences exist in the magnitude of micromotions between the two anchoring designs.?

## Methods

### Specimen preparation and implants

Our experimental model with fresh frozen human specimens for biomechanical testing of the spine has long been established and described repeatedly in the literature [3–7].

The experiments were performed using 12 human lumbar spine specimens (L2-S2) acquired from donors (all males). The segment L4/5 was dissected from the spine specimen as the test segment. The soft tissue around the vertebral body anteriorly and laterally including the anterior longitudinal ligament and periosteum was removed. The anterior longitudinal ligament was resected in the front plane of the disc L 4/L5. The natural disc itself was completely removed and the top and bottom vertebral endplates were cleared of the intervertebral cartilage. Care was taken to preserve the subchondral bone. All other structures of the segment L 4/5 were preserved. The resulting specimens had a regular L4 and L5 vertebral body size to allow a safe anchor of the AcitvL prosthesis.

The ethical standards of the Helsinki Declaration of 1975, as revised in 2000 (5), as well as the national law were respected.

Two specimens were used for the preliminary tests, leaving 10 preparations (mean age: 36.4 years, 18 to 48 years old), selected to largely exclude possible orthogeriatric metabolic bone disorders (e.g. osteoporosis), for the actual experiments. These were divided into 2 equal groups of 5 samples for the implantation of the keel and the spike anchoring ActivL prosthesis (B. Braun/Aesculap, Tuttlingen AG, Germany).

### Implantation

**Fig. 1:**
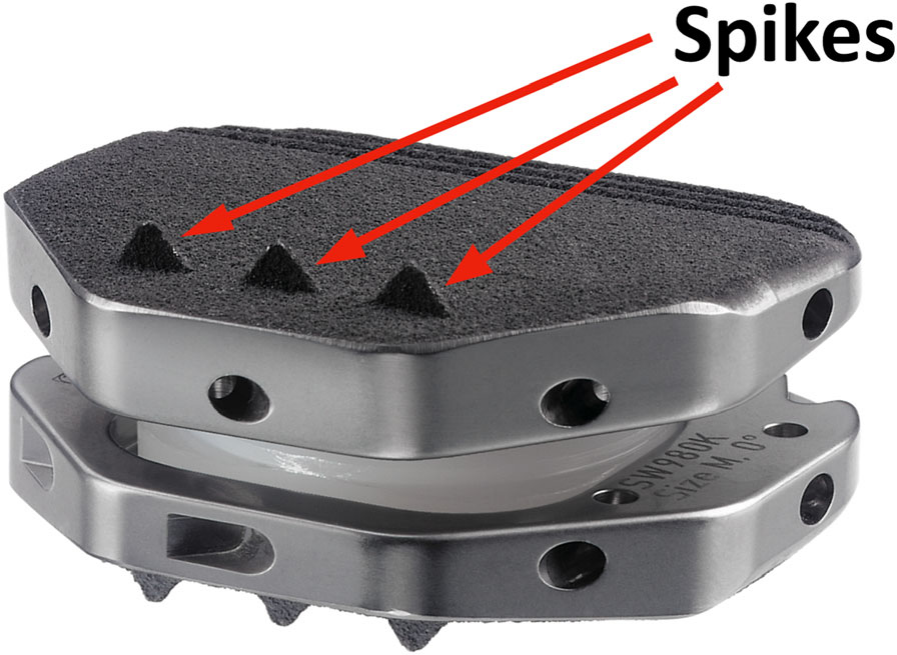
Prosthesis with spike anchoring concept

**Fig. 2:**
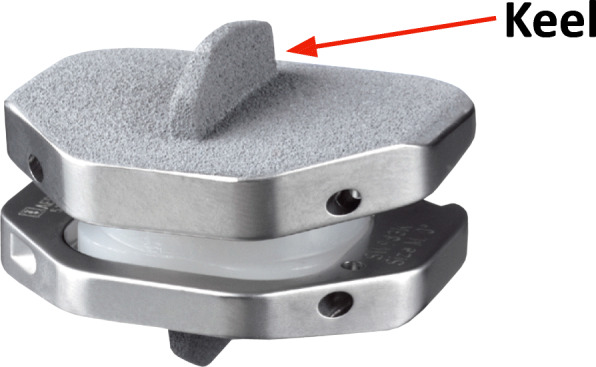
Prosthesis with keel anchoring concept

The ActivL prosthesis consists of three components and is available in two versions. The semiconstrained design allows a limited translation of an ultra high molecular weight polyethylene (UHMWPE) inlay in the sagittal plane. The implant endplates are made of Cobalt Chrome (CoCr) alloy. The spiked version (Fig. 1) owns three spikes in a row at the front edge. The keel of the second disc version (Fig. 2) is aligned in the prothesis midline in the antero-posterior direction. Controlled translational motions of the core in the antero-posterior direction let to a displacement of the rotation center, physiological approximation and normal mobility.

The prosthesis was implanted by only one experienced spine surgeon with a proper surgical technique using the original instruments provided. For the experiments, size M protheses with a superior plate angulation of 6° and a polyethylene (PE) inlay of 8.5 mm or 10 mm were used in all specimens to allow the comparability of the results and to exclude the potential influence of implant sizes.

In combination with the described selection of the specimens a nearly anatomical reconstruction of the motion segment was achieved. In particular, the height of the intervertebral disc space was meticulously reconstructed during the implantation. The lordosis angle adjusted itself according to the anatomical conditions and the current position of the mobile segment. Therefore, it can be assumed that the obtained measurement results correspond to the situation in vivo.

### Experimental procedure

**Fig. 3:**
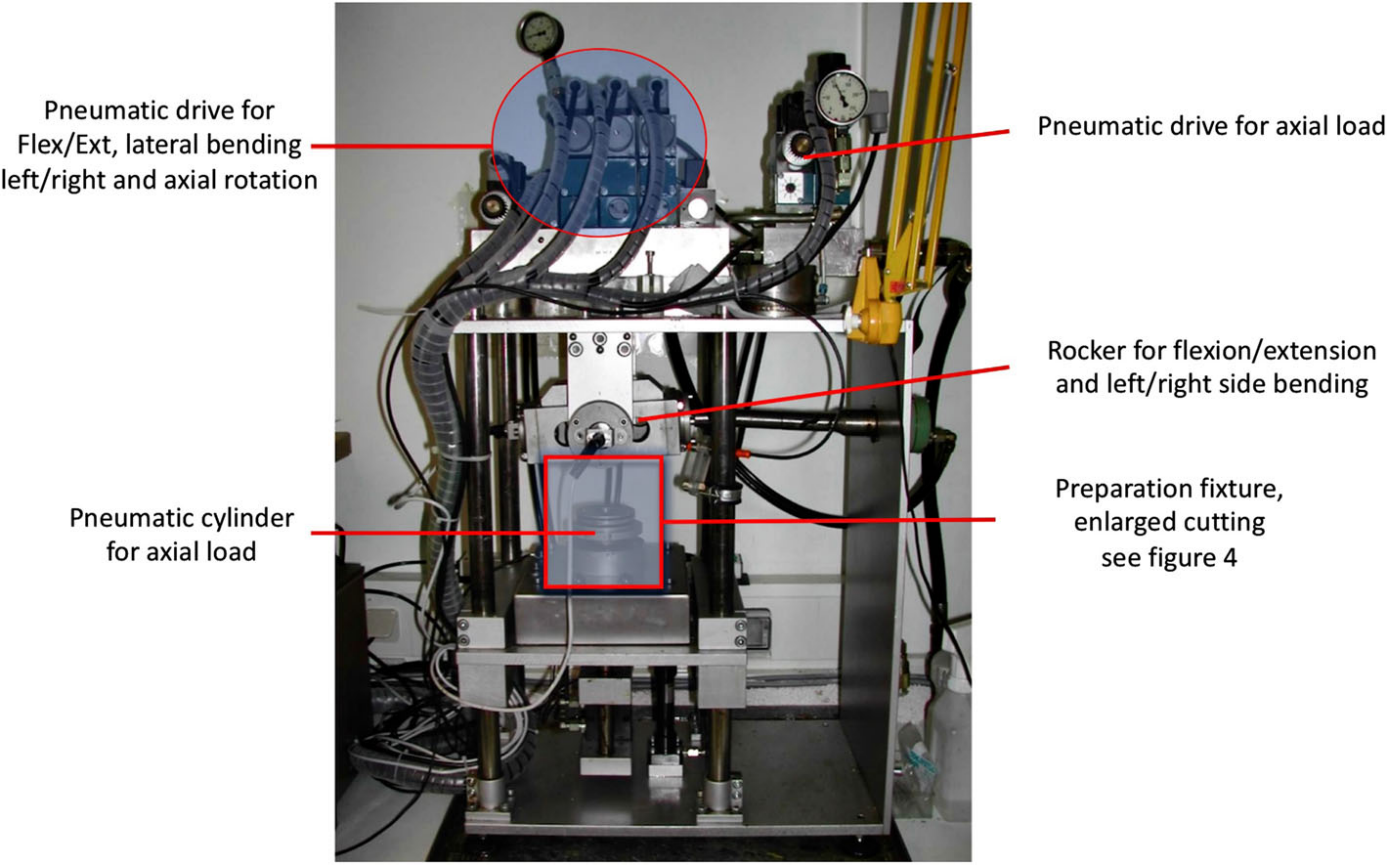
The spine simulator of laboratory for Biomechanics and Experimental Orthopaedics of Ludwig-Maximilians-University in Munich

**Fig. 4:**
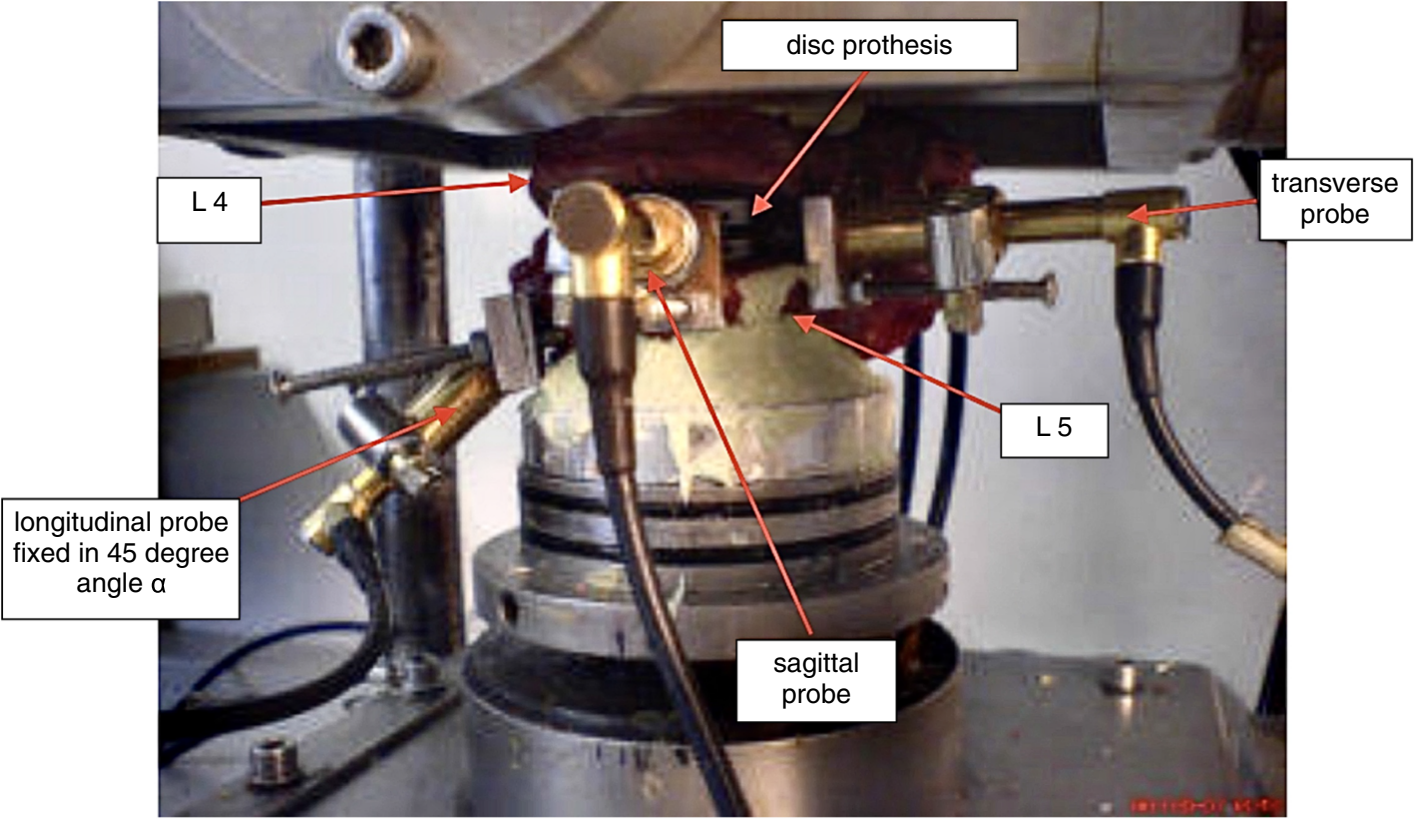
Preparation fixture, enlarged cutting from Fig. 3. The intervertebral disk prosthesis is implanted in the prepared motion segment L4 / L5 that is fixed in the simulator with bone cement. The measuring probes are attached to the caudal prosthesis component. The results of the 45-degree-angle (α) fixed probe for the axial micro motions were trigonometrically converted by the cosine α

**Fig. 5:**
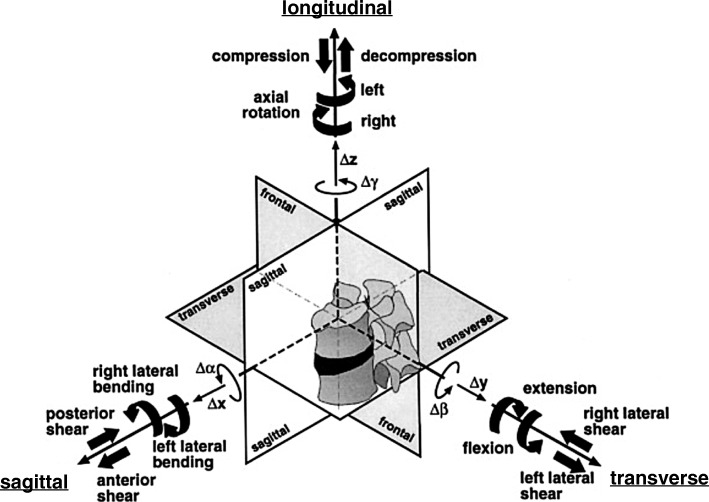
Three-dimensional coordinate system of the spine simulator (according to ISO 2631). All possible load and motion components are illustrated (3)

**Fig. 6:**
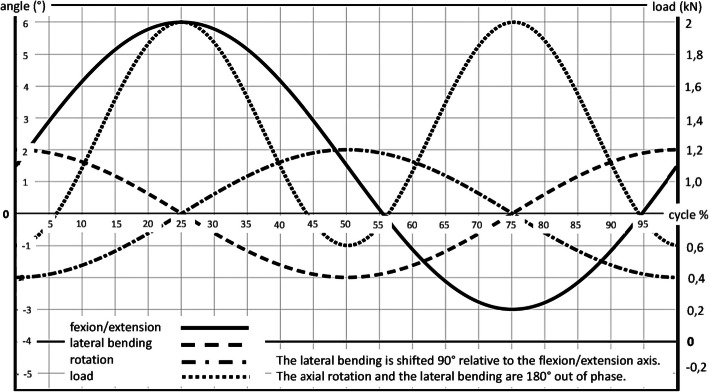
Phasing of the displacement and load curves (alternative) for lumbar prothesis

The experiments were carried out in the laboratory for Biomechanics and Experimental Orthopaedics of Ludwig-Maximilians-University in Munich. An existing simulator (Figs. 3 and 4) was used, which consists of three major parts: a motion simulator, a control block and a connected computer. The motion simulator allows the simulation in three planes with simultaneous axial load. Thus, there occur six “true moments”: in the sagittal plane flexion and extension, in the frontal plane left and right lateral-bending and in the transverse plane left and right rotation [3–5]. The simulator complied with the requirements of DIN ISO 2631 [8, 9] for the testing of spinal implants (Figs. 5 and 6).

Specially attached measuring sensors (Induktive Economic Displacement Sensor WETA 1/2 mm, Hottinger Baldwin Messtechnik GmbH, Darmstadt, Germany) were used to measure micromotions, with a precision of 1/1000 µm. Sensors were attached to the specimen in three coordinate axis. The holders for the specimens were aligned strictly in the sagittal and transverse axis on the L5 vertebral body so that the sensors touched the caudal plate of the prosthesis. Because it was not possible to install a sensor in the longitudinal axis, this was attached lateral at an angle of 45° with the measuring sensor touched the caudal surface of the prosthesis plate, and the axial micromotion calculated by projecting this measurement to the vertical axis.

The final construct with the implanted prosthesis and the sensors was then fixed with bone cement to the special holding device designed in the motion simulator (Fig. 4).

Tests were carried out according to the ISO 2631 standard for defined three- dimensional coordinate systems. The movement areas were set using default values according to ISO/CD 18192-1.3 (Figs. 5 and 6) [10] and are presented in Table 1. The simulation of the natural movement sequence in lower lumbar spine was performed analogue to the physiological conditions (Fig. 6). The simulator tested in each movement plane (sagittal = flexion/extension; frontal = left/right bending; transverse = left/right axial rotation) with the frequency of 1 Hz. The different axes were not coupled.

**Table 1:** Range of motion (ROM) and values of the axial load according to ISO/CD 18192-1.3 for a motion segment in the lumbar spine

	Flexion/Extension	Lateral bending	Axial rotation	Axial load
Maximum ROM	+ 6^0^	+ 2^0^	+ 2^0^	2000 N
Minimum ROM	-3^0^	-2^0^	-2^0^	600 N

The data were recorded via the measuring sensors connected to the receiver module. Processing and presentation of the results was done by the Catman software ® (HBM Germany).

**Fig. 7:**
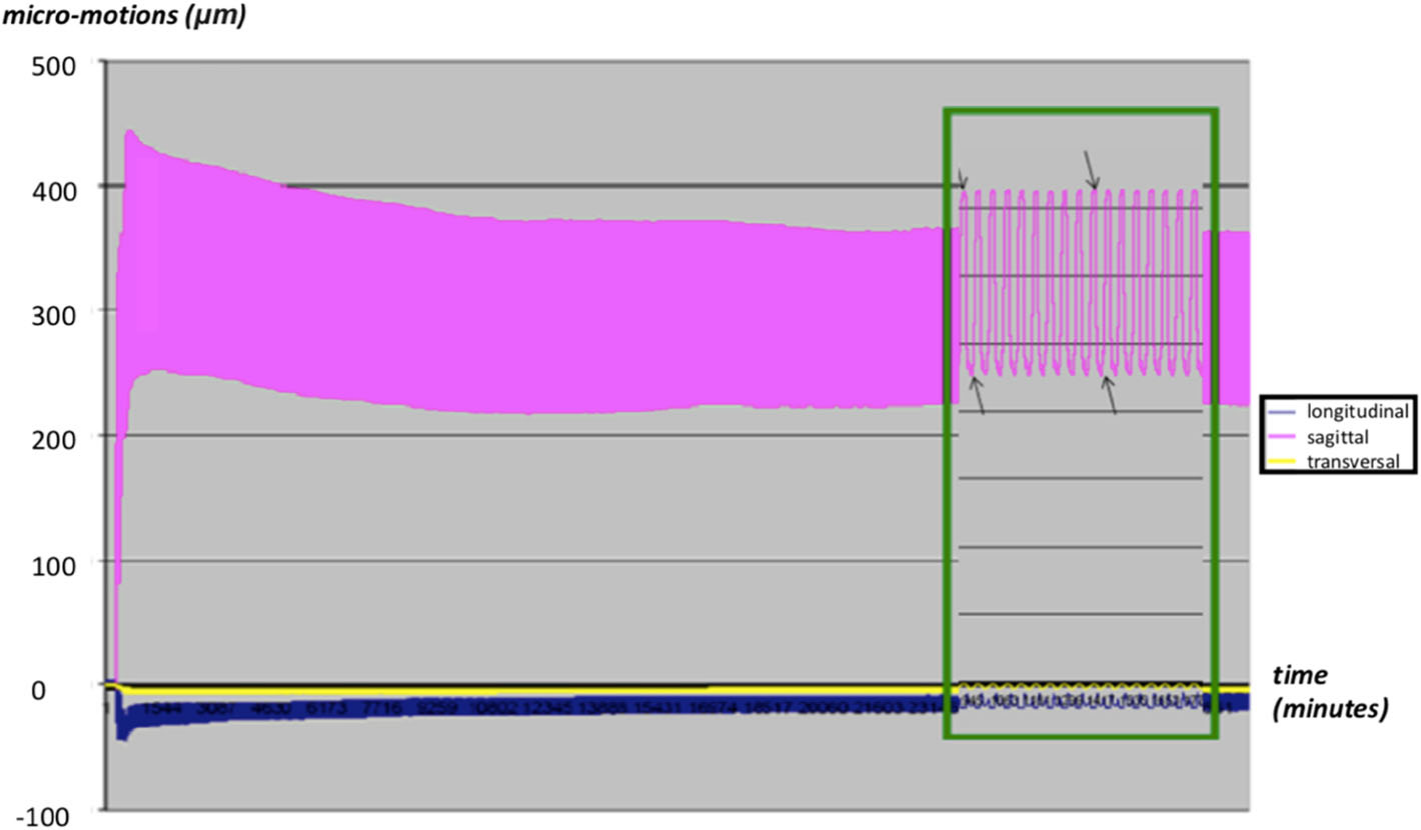
The graphical representation of the measured values showed the stabilization of the measured amplitudes after 400 cycles. In the phase between the 540th and the 600th cycle, 60 representative cycles with 3000 values were selected for the evaluation of the results

Each specimen was tested for at least 1000 cycles (mean 1050) while micromotions were recorded at 50 Hz in all three axes. As micromotions showed stabilization at about 400 cycles, 60 representative cycles between the 540th and the 600th cycle were selected for the evaluation. (Fig. 7).

### Statistical evaluation

Representative mean values were calculated for the prosthesis movement from the determined values for each measuring axis. Therefore for both of the two implant types all representative peak-to-valley-values were grouped according to the measuring axis in an Excel spreadsheet and were fed for statistical analysis in the GraphPad Prism 6 program. For both prostheses, the mean peak-to-valley-values for each measuring axis were calculated from these data.

For the further statistical analysis (IBM SPSS 25.0®) methods of descriptive statistics were used. Data was tested for normality using a Kolmogorov-Smirnov normality test, after which a Student’s T-test was performed to investigate the significant differences in micromotion in each measuring axis for the two anchoring types, with a significance level of 0.05. Mean, Median and interquartile range (Q3 minus Q1) were used for the presentation of localization and dispersion. The median represents the movement level. The interquartile range defines the motion profile.

## Results

In the Kolmogorov-Smirnov normality test all imposed data followed the normal distribution so that the further evaluation was carried out with the Student’s T-test.

Descriptive statistics of micromotions are presented in Tables 2, 3 and 4 for the transverse, sagittal and longitudinal axes, respectively.:

**Table 2:** Micromotions of the prosthesis in the transverse axis in µm

Anchoring Type	Mean	Median	IQR^a^	Std. Deviation	Std. Error of Mean	Lower 95 % CI	Upper 95 % CI
Aesculap Keel	4.65*	4.80	4.48	2.29	0.93	2.25	7.05
Aesculap Spikes	15.85*	15.65	5.85	4.60	1.88	11.03	20.67

^a^*IQR* inter-quartile range

**Table 3:** Micromotions of the prosthesis in the sagittal axis in µm

Anchoring Type	Mean	Median	IQR^a^	Std. Deviation	Std. Error of Mean	Lower 95 % CI	Upper 95 % CI
Aesculap Keel	**39.97**	39.20	24.10	12.79	5.22	26.55	53.39
Aesculap Spikes	**45.75**	42.95	15.20	7.73	3.16	37.64	53.86

^a^*IQR* inter-quartile range

**Table 4:** Micromotions of the prosthesis in the longitudinal axis in µm

Anchoring Type	Mean	Median	IQR^a^	Std. Deviation	Std. Error of Mean	Lower 95 % CI	Upper 95 % CI
Aesculap Keel	**157.00**	155.40	21.90	11.37	4.64	145.00	168.90
Aesculap Spikes	**141.40**	135.10	73.98	42.70	17.43	96.55	186.90

^a^*IQR* inter-quartile range

In both anchor types the value for micromotion were below the required threshold of 200 μm. The keel anchoring system showed a smaller mean micromotion value of 4.65 μm, compared to 15.65 μm and the difference was statistically significant * (*p* = 0.003).

In the sagittal axis the micromotions values of both anchoring types lay also well below the threshold of 200 μm. The keel anchoring system again showed smaller micromotion, but on this occasion it did not reach statistical significance. (*p*-value is 0.365).

In the longitudinal axis the highest values for micromotions were observed, which lay in any case below threshold of 200 μm. Here, the spike anchoring concept shows better values, but a greater dispersion, as shown by the standard deviation and interquartile range values. The p-value is 0.408 indicating that difference is not statistically significant.

**Fig. 8:**
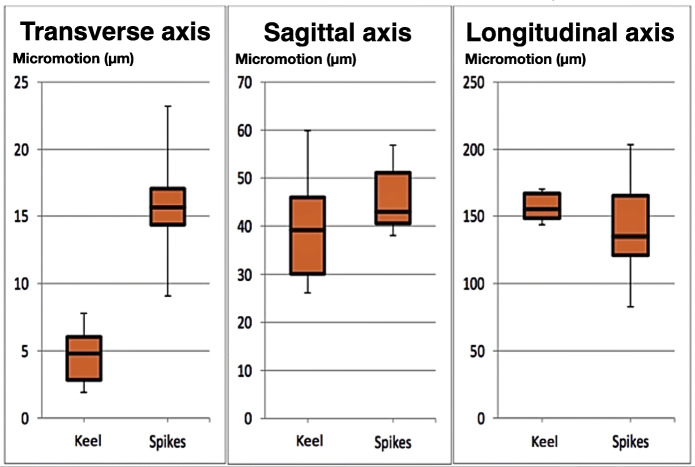
Range of motion of the prosthesis in µm sorted by the three different axes of motion

In Fig. 8 are presented the motion ranges of the prosthesis in every axis in the form of box plots.

## Discussion

To determine the primary stability of intervertebral disc prosthesis with two different anchoring concepts – keel and spike anchoring - ActivL intervertebral disc prosthesis (5 x keel anchoring, 5 x spike anchoring) were implanted in 10 human cadaveric lumbar spine specimens and tested in a spine movement simulator. The micromotions of the implants in the transverse, sagittal and longitudinal axis were determined. These results are a parameter of the primary stability. Both types of anchors met the given criteria of primary stability, independent of the selected anchorage type.

The in this study used biomechanical model for testing spinal disc prosthesis implanted in fresh frozen human specimens is well established. The technique was described repeatedly in the literature [3–7]. Because of better comparability and to exclude early degenerative changes we used only male donor specimens of young age. Therefore osteoporosis and its potential negative influence on micromotion due sparsey structure was ruled out. Nevertheless it has been shown that the mechanical behaviour of spinal segments in the simulator remains unaffected by degenerative changes [10]. In addition, osteoporosis is a surgical contraindication for implantation of intervertebral disc prosthesis [11].

The axial load and adjustment of motion range of a spinal segment was performed according to the ISO values. However, there exists in the literature a recommendation to conduct the experiments without the axial load [4] because outherwise the data may lack of comparability due to the great individual variation of the biomechanical characteristics of the human spine.

In our experiments, the axial load was adjusted between 600 and 2000 N. In the living body values of 2000 N are achieved only when lifting weights of 10 kg or leaning forward with simultaneous rotation of the upper body [5]. Such values seem unlikely in the direct postoperative phase in vivo due to appropriate therapeutic instructions in newly operated patients. This load-adjustment probably led to the observed increase of the micromotions in the longitudinal axis. Theresults did not show a statistically significant difference between the two anchoring concepts in this axis.

The spiked prosthesis has shown lower mean and median values, but greater dispersion of the measured values. On the other hand, the keeled implants showed more homogenous results, but nonetheless high micromotion values. Both concepts guarantee a safe osseointegration because complex multi-axial movements combined with axial load, as mentioned, are not expected in newly operated patients.

In the sagittal axis the primary stability is given with both anchoring concepts. Our data showed no significant difference between both groups. The statistically significant difference, which was observed in the micromotion values in the transverse axis, gives a slight technical superiority for the keel anchoring concept and validates our hypothesis that differences can exist due to the anchoring type used. This effect was expected due to the larger contact area of the keel with the bone, which also provides a larger area for osseointegration. This advantage has only a relative clinical importance, because the observed motion ranges of both concepts lie, with exception of the longitudinal axis, very well below the primary stability threshold of 150 μm postulated from Jasty [1].

Bah [12] and O´Rourke [13] report in their current publications on cement-free hip prosthesis (Furlong Evolution cement less short stem, Pinnacle Cup) about calculated micro motions well over 150 micrometres. Nevertheless, the Swedish hip arthroplasty register [14] reports excellent long-term results for the Pinnacle Cup. It can be concluded that a safe osseointegration of cement less implants is possible even in micro motions well over 150 micrometres. Pitto however postulated an osseointegration of cementless implants up to micromotions of 200 μm [2]. All prostheses examined in our study fell well below this limit of 200 μm, even in the longitudinal axis, which validates our first hypothesis. The measured micromotions in this study above 150 μm, but under 200 μm in the longitudinal axis seem to be without relevance in surgical practice.

Jasty [1] found osseointegration of the implants in his histological examinations of dogs 6 weeks after implantation of cementless implants if micromotions of 150 μm were not exceeded. After 6 weeks the process of osseointegration is not completed as indicated by scintigraphic examinations showing accumulations at the bony implant site over a period of 2 years [15].

Current clinical publications [16–18] confirm that the ActivL disc arthroplasty is a safe and effective procedure at least in a short-term two-year follow-up. The cautious conclusion suggests that the implants were successfully osseointegrated in this period of time.

The graphical representation of the measured values showed the stabilization of the measured amplitudes after a passage of about 400 cycles. This is due to subsidence of the prosthesis in the early phase of the experiment. Conclusions on additional subsidence of the prosthesis in vivo in the context of osseointegration are not possible based on this cadaveric study setup and can only be determined by imaging procedures on living patients.

The rehabilitation programs developed for the acute postoperative phase focus on stabilizing exercises strengthening the autochthonous back muscles. Lifting, twisting and hyperextension are prohibited. It is known, that the highest stresses and therefore probably the highest micromotions in the disc tray, arise in combined flexion and lateral bending under axial load. Our results show that after the implantation of the prosthesis with both pegs and keel, the primary stability as prerequisite for safe osseointegration is provided. Based on our results, we see intervertebral disc prostheses as an alternative to fusion operations in the lumbar spine in special cases.

## Conclusions

Artificial disc types anchoring with spikes as well as with a keel meet the criteria of primary stability regarding micromotions below 200 μm. The keel anchoring prothesis showed statistically higher primary stability compared to the spikes model in the transverse axis.

## Data Availability

The datasets used and/or analyzed during the current study are available from the corresponding author on reasonable request.

## Abbreviations

Hz: Hertz
ISO: International Organization for Standardization
ISO/CD: International Organization for Standardization/ Committee Draft
µm: Micrometer
